# Supplementary material for: GmWRKY81 Encoding a WRKY Transcription Factor Enhances Aluminum Tolerance in Soybean
Source: Int J Mol Sci. 2022 Jun 10;23(12):6518. doi: 10.3390/ijms23126518 (PMC9224350; doi:10.3390/ijms23126518)
Supplement: Supplementary file 1 [file ijms-23-06518-s001.zip › Supplementary Data.pdf]

Supplementary Material

1 Supplementary Data

1.1 Supplementary Data S1

CDS sequencing information of *GmWRKY81*

ATGGCCTCAACCAACCATAATTCTAGTTTCTCTCATTCCTTCTGTCCCTTAAT
CGCGAAGCAAACCTTAATTTCTTCCATGGGAATGGAACCAACGTGCGTCGA
CACTTCGCTCAACCTTAACGTTATTCCCTCTCCACACATCGCGGAAGAAGT
TTTGGTTGAAGAGTTGCGGCGTTTAAGTAATGAGAACAAGAGATTAACTGA
GACGCTGAAACACGTTTGCGAGAACTATGTTGCTTTGCAGAAACATTTAAA
TGAATTCAGCCAACTGAGGAACGCAAATTTTGATAAAGAAGCAGGAACAG
TACCGTCACTGAAGAGAAAGGCCGAGAGTGTGAATCTGTTTGGTATTAACA
ACTATACTGAGTGCAGCACCATCACTGAGGAAGAAACATTCAAAAGGCCC
AAGCATAGCACCGAGCCAAAGGTTTCCAAAGTTCTTACACGAACAGATGC
ATCTGATACCGGCTTATATGTGAGGGACGGATATCAGTGGAGAAAATATGGT
CAAAAAGTCACCAGAGATAACCCTTCTCCTAGGGCTTACTTCAAGTGTTCA
TATGCCCCAAGCTGCCCAGTGAAGAAGAAGGTGCAAAGAAGTGTAGAAGA
TCCAAGTGTGTTGGTAACAACTTACGAAGGAGAGCATAACCATGGGCAATA
A

1.2 Supplementary Data S2

Promoter sequencing information of 15 shared genes

Glyma.02G157900

TATAGTTTAATATTTTCTATTCTTCGTAGCTCAATTGACATCTCTTTGATTCTT
ATTTTCTCACCTTAACTGAGTTTTGGTCTAAGGCAAATGATTGAGTTTAATT
GATAATTGTAGCTTAATTTTAGATTTTGTGCTTACTTGACCTATCTGTCTTGT
GCAGGGCTTAGATGACACTATAGAACTCCAAATGGAATGTGTAAATAGTCC
CTAGAAAGATAAGAAAAATGATATTAAATTTTGTTACAAATAATCTTTTTCC
AATTCTGCCATTTCGAGGATCAAATTGAGCTTAGAAGACAGAGCCTTTGCA
CTTGAAGAATTGGGCTACGAGTCTAGGCTTTTGTTTTATGTAATTAGTTTAG
TTAGGTAGTTAGTTAGTTAGTTACTAACACTCTATATATTAGTGTTAGATAGTT
AGTTAAGAATTGAACTCCATTTTGGAGCCTCCTAGTTCTCTTTCATTTGTCG
TCAACCTTCCCCTTCCTTCCACCGTTCAACCACCTTCCTTGGTGCTGAATTC
GCTACCTAAGTACCTTAAGTATGCAAAGTTCTTGAAGGATTTGTGCACTCAT
AAGAGGAAGCTTAAGGGAAATAAACAAGTGAAGATGTTTCATGACCCTAA
ACCTTATATTGTCCAACAAATGCTCTTGTTTAACTCTCACCTCAAGCTTATTT
TCGATAAACTTCGATCTAGATGGGATGCTTTTGTTATTACTAATATTTTTTACC
ATGGTGTAGTTGAGATTAAAAATGAAGTTATTGGAAAAATATTCAAAGTGA
ACAGTCACCAACTCAATAAAATCTTCAAGCTTTTTCATGAGAGCCCCTAAG
TAGAGGAGGAATTTGTGGGGAACCTCTCTTTGGTCTTGCCACCAATTTTATG
TTATGATGTCCCTTGAATGGCACTTGAGGAATTTCCTTTCCCTTTTCTTTTAT
ATGTTGTTCCTTTGTTTGCATTTCATTACATGCTCACATTGAGGACAATGTGC

ATTTCAAGTATGGGGGGAGGGTTTAGAAAAATTTGTTTTCTGTTTTTCTATT
TTGCTTTATGTTTTCTATTTTGTTGTATTTGTTTTGTTGTGTTGTTTTACTGTT
TTTACATAAAGGAAAAAAATTAGTTCCTGAAAGTGTAAAAAGTGTGACAAA
TATATCCAAATATTAATAGTAGATTGTGACTAATTATTATAATTTTGAAAAATT
TATATTGATTGCGGCAAAATATTATCAATGGAAGAGAATTTTTATTGCTTTGA
CAAATTCTTCTTACTTTTCTTCAACTATAAAAAAAAATCAATCATTTAATTGT
TAACTCTTGAATAAATGCTATCAACACAAAAAAAAAGAAATTTTACTGTAA
AACTATAAGAAAATCATTGTTTTTGTTTAATCAAACACTATTTATTTAATTGT
TTTTTTTATAAATTTTTCATAATAAAATATGAATGTCTATTAGTATATGCAATG
ACATCAAATTACAAATTATAATAAAAGTTCGTTGATTTTTAAATTAATTTTTTT
ATATCATACACAATTATTTTTTTTAACTAATCATTTAAAAAATCATAACCTAAA
AAAAATCATTTCAAGGAGGTGAGTGTTTTTTACAAATTAAAAATGTTATTGC
AATTATAATTTTTTCTAAAAAATTATTTAGGGATCTAATTTAATTATAATGCTT
TATAATAAATATTTTTTTTACTTAAACCTTTCATCAAGCATAAGAATATGAAA
AAGTTATTTACAAGTTTATAAAACTAGTATTCTTCTTTCTTTTTGATTCAATTT
AATTTACATGTTTGTTAAATTTTTTTTTATTACAATCCATATAAATTCGATATG
ACCACATTGACAATCATTTTGATGACACATAGGTTATTCTATTAATAATGACG
GACAAAAATTAATAATGAGATATATTTATTGTACTTTTTATATTTTCGTAGACT
AAATTTTATATTTTTAGTGATTATTTATTTTTTTTATTTTTTTTTCCGGAGATGT
ATGAG

Glyma.03G130600

TTTGGTTAAGGGATCATATACTAAAAGGTTTTTAGTATTTAAACGATGGAAG
ATATGAGTTTAAATCTTTGATTGAAAAAATTAATAATTAATAACTAATATTTAT
TATTGATAAAAAAGAAGTCAATGAGTACAAAAAGAAGAGAGACGATCGAC
AAAGACATGTACATACGGACGTGGGAATAACTTGTATTTAATACAGTCATCT
TGGTTAAACGACGTATATATTGTTACTAACTTCAAAACTGCTTTATCTAGAA
AGTTAGTACTATTATTATTTATTATCATGTACTACCTAATATAACTGACGGATAT
AATACAACTCTACTGGGTCTTGCATGCATCATCATATTTTGCTATGTACGCTA
AAATTTAAAATTCCCAGCCTTAAATAATAAACCCGTGCAAACCATAGGCAA
GGAAGCCGAAGGCTTGTGTATAGCATGCATCGGCACGTGCAATTCAAAGAA
AACAAATCGGTTTGCCTACAAGTGCCTGCTTATGACATATTCATATAATTATT
TGGTGTGGCCAAAATGTTTGCCATTTAGATTAATGCATTTTTATCCTATAAAC
TTTTATGTACAACATGATTTTAAATCGCAACACGTTATTATTATTACAAAAAA
TATATGGATTTTTAATAGAAAATATATAACTTATGCAACTGTAATTGGATTGAA
TTTCACCATGCAATGGAAGTTCGTATACTCTTACCCAGTTACCCGCACTTGT
TTTCTTCTCATTTCTTTCTCATTTGAATGCCATCAAACAGAGCATAAATGTAA
GTTGTGACTAGTTTCAAATTAAAATTGGTGTTGTTACTTTCAGCTTTTATATA
TACCAATACCATCCCTTTTTTCTTAAAATCCCAGCTCCATTTTTTGCATCGTT
GACCAACTTTTCACTTAAAATACATATAGCAGAACCTAATTAAGGTGATCTT
CCAGTGCAGTGATTTTAAATTACGGTCCGTAACCTTAAGGTATTTTTTCGCA
ATTACAGCTACATCAGCCACATTTCTCACAATTATCAGTAATGCAACTGTTT
TCTAGTTTATCGCAACCGCAATTTAAAATAATGCAGGACCTGAATATATATGG
AAGTGTGTGTATGAATTTTCAAAGTGGTTCACTATATTTGTAAACACAATCA
GATCGGTTGATATAAAAGATAAAACATGTGGAAAGGGAGAAAATTAAGAGT

CGCAACCAAAACATGTTAATGTAATCAGCGATATCGTCTACATCTCAAATAA
TGTAGAAAATGCTTCTGAAAGGTTAGAATATTGGGGTGGATGACATGTGAT
ACTAATTAAAGGCTATAATTTGTTTCTTTTTCTTCTGCAAGCGTTCTTTTGTA
ACAAGGGCGAGGCAGTAAATGCATTTAATTTGTGGCTTCTCTGCAAGAAAT
TAACTAATAACCAGCCAGGTTTGCATGGTTATTATATTGTCTTATTGGCTCCA
TTTAATCAGCTACAATTGTTAAAATATTTTTAAGACTTTTAGCAATAAACTTC
ATTATGTAACACTTATGTCGTTGGCAGCATAGCAGAGAATATACCAATATACC
AAACTCCACACATTCATGAAGAAAAAAAGGGATGCCACATAGACAAATAA
AGAACTAGAGAAATGTATCGTGTTTAGGTTTTTCATACACATGCTCTCCCAC
CTTCTCACCCTCTCTGCTACATAGACAAACACGTAGATGCCACATTTCAGAC
ACGCTTTTGCATGTGCACCTATATAGTTCTCTAGAACCAAAATTATTTTATGA
TGATGGATAGTAATCCTGCAATTATTAAATATCTTAACTCCTACTTGAAAGAT
GAATTTATATTTTGAATATTCCAAGTGACGAGGAGAAACAAGAATACTTTAT
TGATTTTTTATCAGCCACTTATTATTTGTTCAAATATATAAACATGCATGATTA
CGTAAAAGGATATAATGACTTGGGTATATATATATGTTCTTATTCACAAGTCG
AGATCATCATAGCATTATCTAAGTAATTTGTTAACTGCTCCTAAGTCCTAATT
AACAAATCTTCATCATCATCATGGTTGCT

Glyma.04G035600

AACCCTTTTTCTTTGTCACTTAAGCACTCCTCCTACATTATATGGAGGAAGA
GAGCAACTATATATTCGTATGAATGATTTTTTATCCATGATATATATATATATTT
ATATATTTATTAGTTTTGTCTAACTATAAAAAGAGATGTGAGAAGAGGATAA
ATCTTTGCTAGGAAACATTGGCTTCTGTCCAAAGAATTCAGAATATGTCTTT
GAAGTCATTTTCAGTGCAAAGAATAGAAGAAATTAAATATAATTGCTCTTTC
AGATAATGGACCATATGTTGTAATTGTTCAGAAGAGGACCCATGTTGAACA
GGCCAGGTGGGTCAAGATTTATGCGAGACTATCATGACATCATTCATTCTCA
GATCATATTTCATATTTGTATCCAAGAGATTTGTGATTTTTTAAAAGCCATAA
AATTGTTTTTTTTTTATCACAATGTTCAGCATTTTGATTTCCGATTTTCGTCG
GAATTTATTTGGTTTGAAATAACATACTTGTTAAAAATTAACGTATAAATCAA
ATAAAAAAATTCTCAACGAATTTTTTTTTTGAATAATACATATATCTTATTATT
TAAAACATTTCGTGAACATATCTTATTTCTTTTTATCAAATCACAAATCCTAT
CAAACATATATATTTCTCTCTTGGTGTTCAAATGGATGTTAGGAGTCTTTATG
TAATATTTTTCTTGTTTTTTTAATGCCTCAAAAAATTGGTTAAATAGAATACA
TCAAAGTTTTGCTTTTTTAAGTTAAACTTGTCAATTGAGACGCTGAAGAAA
AAAAATCTTTAAGATAAACTTGTGGATTGAGACGGAGATGTACAATTTAATT
ACCACTATCTTTTATACAATTTAATTAAGTACGCTTTTTAAGATGTAATTATAT
GAGTTGAAAATACCCTACATAAATATTAAGGCTACATTTAGATAAAAGTTGA
GAACGTATTTTTAAGGAATTTAATGTACATTTCTGAATTTCATACACAAAGA
AAAAATTCTTAAAAAAAGTACTTTAATCTCATAAAAAATATCCAAACATTGA
CTAACTGAATTGCCGTGATTCATTCTCTAGGAGTGACATGGATACTGAATCA
ATTCTTAATCCAAACACACATTTTTTATGTTAGACTAAATGGTTGACTAATCG
ATGATTTTGTATATTATCTGACCTCGTTGTGGTCATGACATCATCTTATTGTTT
ACTCCGTATCTATTTCTAGCGTTGAATTTTTGGAGCTCTGCAATATTTGCCAA
TATTATGATTTGTGATGATATTATAATAACATGAGCGAGTCAGCAATTAAGCA
TGAAGCAGCCGTGCTTTATGATCGTCAAACTCGATGATGATATTGGGAGGGT

AAATAAATTAGACTACTTGTACCAATGATTTTCATGCGGAAATGGTATTTTTC
ATCCCTGTTCAACATTAATTATATTTTGTTTGGTATGCCATTTTAAGATGGAAT
GGAGAGATTAGATGGATAAAATCTATTTCTTGTTTTGTTTTGGGTGACATATA
ATTTTAGTGATACCAAACAAAACCAAAAAGTTGAGAAACAGATGCCAAGT
CAAGTTGCCTCAATATCATGTTAATTTTGCTTATAAGCATGCATATTTGTTTTC
TCTGCCATACACTATCATTGATTCCCCTGTATACAACACCATTAAACCTCTGC
CATCACTTGAACCATCAATAAATGTAAGGGGAAGAAATAAAATAATTACATA
TGAGGTAGTGGCAGATGTTAGCGTAAAAAGTATATAAAAAATAATTAGATAT
GAGGAAGTGGCATGAATAATGTTGTTTGCAACTTTAGTAGTTTGAACTTTG
AAATACTAAGATATCAGGGACAAAAAATTTGGAGCGAGTGTAAGGTGAAG
GCTGACCTGAAACCATTGTTAAAGACACGGCATGCTTCATGCTAGTAAATT
CAGTACGATTTTTGTATTATAAATTGATCGAGCGCCATCTCCGTTCTGTGCAA
TCTGCTCTGAACCATGGATTCC

Glyma.06G115800

AGTAAACTTAGCATCTGTTCCCAATTAAGTTACGCCCATTGAGTCATATCCAT
CTTTCATCATAAATTTCCAAAAAATTATATTTTTATTGGGTAAATGACCATTTC
AATTCCTAAATTTGTAAACCGATGACAATTTAGTTATTGAAAGAACAAAAAA
TTATGACTTAATCATAAATTTATCATAAAAAATATAATATAATATTAAATTAGT
CATTAAACATTACAAATTAATGACAAAACGGTCTCATGAAACTAACAACAA
GATATAATTGTTACAATTTTTATACATTTACAGACTAAATTAGAATTTTTATTT
TTTAGGGACTAAATTATTACTAATTTATACATTCAATATCAAAATGATCATTTT
TTTTAGAGTACATTACACTCATGTTGATACGAATATTACATTGTGTTTTCCTC
TTATATTAAAAACATTGTTTAGTCTATTTAACTTAATATAATTTAAAAATATTAT
TGATAATTACTCTTTATTTCATGTGAGATACGAGTAATGCATTCATTTGATATG
AGCTCATATTTTTTTTAAAGAAGGTATGAGCTCATATTTAATAATAGTTATATT
TTTTATTTATATTTAATATTGTATTTTAAGCGATTTAATCAAGTGAGTTTGGCG
GACTACTAAAAAAATACAATATGAACTTAATTAATGTGTGAATTTTATTAGTG
ATATTTTTCTTCTTCTGAATATTCTATTATTAGTATACTTAAGCATAATGAGAA
TCCTAACAAAGTTAATAAGAAATTCTAAATATAGTCTCGAATTTATCAAGTTA
CATAATTTTCTCTTTTCCCATTTGAAATTTTTTTCATATAGGAATTAAATTCCT
CGACTAAGAAATAATCATGAATTTACAATGCAGTTATTTATACGAGTTGCTC
AAAAATAAGATCGTGTTGTTTAATAAAAGTTGTCATTATAACATATCAGGTG
ACATTATATATATATATATATATATATATATATATATATATATATATATATATA
TATATATATATATATATATATATAACATTCTCAGACATGATTCAAGCTATTTTTG
TTAAATTAAAAAAATTCTACCCTCGCCACATTTTTCTCACAATCAAGAGAAA
AAAAAAAGAAAAAGAAATATAAAATAAGATAAATGGTATCAACGATAAAAA
AAAACATAAATAATGTCATAAAAAACAAAATATCAAATATAAAAAAAAACA
TCAAGTTACTCCAAAAATAATTCTAGAAAAGTTATTCCTCAATTTTATTATTT
ATCATCTTAAAATATAATAATTAAGATATGCAATAATTTTGAAAAAAAAACAA
TTAGGTGAAAAGATGATACGTGCTAAAGAGAGTAATGAGACATGTGTGGAG
TAAAAATGTGCCAAAAGAGAGAAAAATTTAATAAAAATAAATTTGACCTTT
TATTATGTATTATGCAGTGAATAAAAAGTTTTCTCTTGATTTTGCAAGTTAAT
TATTTTTTTTTCTTTTTAATTTTTGTAATAAGCATGTAATGATTAATTTTGTAGT
TGTTATTTTTTACTTTTAAGCATATCAACCGCTACACAAGAAGAAGAATGTT

TTTAGCATTATACTTTTTCGCTGGAGTTTGACTGATGCTGAATCAACTACTAT
TTGATTTTTCTCTTTTATAGTTTCAATTCATCTTGGTCCTTTTATTAAAATTAA
ATGCTCAATTTTTTTTTTATCAAATTCTTTTATTCAGTTTAATGGGCCAATAA
AAAAGAATAGCAAATTTTGTTTGTTCACTGTGGTTTTGTCCATCTGAACTTG
GATTAATTAAATATTTAATCTAATATCTAAGTACCTGATTCTACACGAAAAGA
AAAAAAGAATAACAGATTAATAAGTGTTCTAAGCATTACATTACTTTTTTCA
CGATTATTTTCTTTAAATAATGAACAGAACTATTAATTAAGGTGAAGAAGATT
AAAATGCCCTCTGACTCATGCTTGCATTGAAGACAGGGACGTGGCTGTTAG
CTGGCTCTTTGTAAGAGGCATGAAGATGGAACACTGACTTGTTTGAAAGCC
AAAGAAGTAGTAAGGATCATCTCTTTTGACTATAAATATGTAAACTTATAAG
CATACTTTTATTCATTCAATCACCTTTGCACTGCACACACAACACTGATAGA
AGGATCAAGGATGGCTAGC

Glyma.07G067700

TTTTGTGAATATATTTATTAAAATTTATCGAGATCATTTAATATATAATTAATTT
AATTATATAACACCATATTTTTTTATAATATTTAATTATAATATATAATAAACTTT
ACATACTAGATTAGATTTCACTCATGCAATACACAAACTAAAATACTAATTA
AGATAGAACAAATTTCTTTACCATGATAGATAAATATAAATGGACGCATACTA
AATACTGATACTTTTTTTTTAAAAAAAATATTACTCTATACTGATAAAAATAA
AATATTTAGCTGTAAATATTTGATGTATTATAAAAATATTTTTAATTTAATAAG
AAATCGTAAATAGTTTTTTTAGGTAAAAAACATAAATATTTTAAATTTAGAAA
TTAATGGATATACACTAAAAAAATACAAATAAAATAATTTTATATTGTCAACC
AAACATAAATCATAATTAATATAATTTTTATGATAGTTATTTTAAAAGTTAATA
AACTTATTTGTGCATATATAATTTTTTATAATCATTATACTTAGGCTCTTTGAAT
CTTCGTCAGATTCCAAAATATTTTACAAGTTTTCGATCCTTTTAATCGATTAT
GAAACTAATTTTAAAAGTCAAGACTTAATTAACTAAAAGTTTATATTCTTTC
CCTAATATAATGGTAAGAAATATCTTTATATATTTTAAAAGAAGAATTAGATTT
AAAATATATTATAATATAAAGATTTACACTTGTTAAAAAGGTACCATTAACTA
AATATTCAGTGTTTGATTTTATTACCATTAAAATGGAATCTATTGGAAGAAGA
GAATTTTTACTTTTATTTATTTATTTATTTTATTACCATTAAATGACAGAAAAT
TTTGACTTTGATAAAACTTCTCTCAAAATGTGTGTTTTTCTAATTTTTCAAA
AAGAATAATTCTAAATCTTTCTACAATATTCTTTTACTTTCTTTTTCCTATGCA
TGATATTTATTAAATGTCTTTTATATTATGAATACATTAAATATATTTTTTATTTA
TTTTTATTGATATTTTGTTTATATGGTAGTAAAAAATTTATAGAAAATAAAATA
ATTTTAAATATTAATATTAATATAAATATGATATGAATATATATACTTTATAATAT
AACATGTTATATACAGAAAAAGATTTTATTAATATAAAAATATTTCACCGCTT
AGTTAATGACATCTTATTTTTTTAGAGACAATAACTGAGTAAATTAAAAGAA
AATATTATATAAGCAAAACTATTAATCATAATGTATTCTTTACTCAGCAGCAC
ATTTCCACATGCTTCAGAAAGGCACTGACCCAAACATGGTTTCAAACAGGC
ACGAGCATCGCATACATGCTATGAAAAATATAATCCTTATTAACCTCAAAAA
TCATTGATATGATCAAAGTATATATAGACAAATACAATCATATATCAAAGCTTT
ATGCAAACAGAAGCTGATACATCCGTTAGTTCTAACTAGTTGGGACTTGAA
TTTAATTTTTAGATTTTTCCATACACAAATTTCACTATAAATATTAGTTAAAAT
TGAAGGTCGTCTTTTTTAGCCTTGCTGTGTCTTCTCATATTCTAAATATTTGT
ATGTACCCAAAAAAAAAATAAAAAAATCAAAGCTTTATTTATTTATTTATTTAT

TTGTGTCTAGTGGAGTGAGGAGGGGGTTCAAGTTGTGCTTATTTGCTTCTC
TCATTCGGCATTATTCACAACACTGAAATGGTTGTTGACCTTTTGATTCATG
TCAACTCTATGTGACCCACTTAATTTGGGCCCACAAACTATGATGAAAAAA
ATATACTACTAATTTCTACATTCCTAGGGGAGGGACATGAAGCATGCATTTTT
GTTTTTATCCTTGTGGTGGTGTCCCATGCCTAATCATTTATATCTGTATAATTA
ATTTATTGTCCAATAACAAGAATTACATTTCTTCAACTATAAATAGGTACATT
CCACTGAGATTCTCCTCACTGCAATCTTCTCTTTTCTTCACACAAACTTGCC
ACATTGTGCCAACCAAAAAACAAGATGGCTCAA

Glyma.08G175400

AAGAAGAAGAAGAAGAAAAAGAAGAAGAAAAAAGTGTTAACACATGCGT
ACCAACAAAATAAGTCATATCCAACAAAAAGTTGGGTTCATGTCCAATTAG
ACAAAAAAGAAAAAGAAGGAAGAGTGTTAAAATATTTTCAAGTTAAAAAT
GATATCATGTTTTTTATGGAGTTAAGAGGAAGAAGATAAGAATATTTAAGAC
ACAAATTTTCATTAAAAATTATGTGACCAACATATGCCTACTTCCTGTTAAAT
TATTGAATGATGTTAATGAAGGAGAATCTCGATGTAATGTGAGTTAAACATA
TTAAAAAAAAATTCATAGGATGCAAAAAACGAAGGGTATCAAATGCAATTT
AAAAAAAATACGAAAGATGCGAATATAATTTACTCAAAGATAAATAAATAA
AAATATATAAAAATGTGGAGTGATATGTGTATCATTGTCCTTTATTAATACCTT
TTATATTTTTATCTCTCTTTTCTTATCACATCATATTACTAACTATATCAATTATT
TATCTTTTTTTTCCTTCTTATCTCTCTAAAGATATAAATACGCCAAATTCAAA
GAGTGTCCAAGAATAAAAAAACTTTAAAAGACTCATGTTAATATTTGTTCG
AACTTTGCTTAAGTTCACCTATAGATGAATTTAGTTTCAGTATACTTACACGC
GTGGTGATGCAGACACCCCTAAACTTATTGATAAATAGCATAGCATGTGATA
AAAAACAATCTTATACTCTTGTCTGATTATTAATTTTATGTTTTTTTAGAAGAT
TAATTTTATGTTATTCCTTTGTTTTTTATATATTAAAGTATTGTTTTTTCAAAGA
TCTTCAATTGCTGACTTGCATGCGCCATGCCGCAGCAGGACTTCAAGCCTAT
ATAATATTCACCTTCTCGTATATAATGACTTAGTGTGAACACAGATATGCTTC
CACCTTCTCATTCTACAGAAGGTGTGTGAAAAGAACTTAAGATACCATCTC
TGATGATCAATTAATTTCAGTAAATTTAATAGTGAGAGACTTGCAACATCCA
CACGCCTGAAGGTCCACTACCCCAAATAGACAATTTTTTAATCAATGCTTTA
TCACAATTTTTTTCGTAATATTTGTAACCAACCACGCACTGTATTATGATTAA
TTAGGATTGGAAATAGATTGAATTAAACTAAACTTTAGACACGCAATTCTGT
TAAATTAAATATTCGAAAAAAAATAACTTTAATTGTCTTCATATGGATCACCAA
ACTTGTTACTTAAACTTTATAAGATTTGAACCAAACCTATTACTTAAACATAA
GGTACGAGTCTAACTATTTGTTTGATATAATAATAATAATAATGGATGTTTAAT
AAGCATGTAGTCTAAAAGATATATATTGGTATCTACTCAAAATCATTCTTGAT
ATAATTTTTGAAATAATGATATTATAAAAGTTAATAAATTTATTATATATAATAA
TAATTTATAATTAAATAACGTACGGCATAAGGATGACTACACTATTTGAGTAT
ATTGTATGTATTATTTATTCATATTATTAAGGGAAATAATAATTAGCATCACAC
CGTTAATCAAAAATGAAAAGTTTATATTAAAATATATTTTTCCCCAGCACAGT
TTCCAATGTATTGATAGCTAGTATAATTTCTAGTAAAAGCTCAATATATTAATT
TCTCAATACGTACCTTTAGGATAATAATAATAGTAATAAAATAAACGGACTTA
TTAATTTCTTCCACCTTATTTGTGATGTATATTTTGTGCAATTCCAATATTCAG
ATGATGATTAGATAACACCTATATATCTATCACCATCATGTATCCAGGTGATTA

CAATCTTGAAGGCATGAAACACCCCTTTGTAGTTGACCAGTACATGGATTT
CAGAATTCCAACATCATTAGACAATGTCCAACAATAACAATAATGACCTTTC
GATCCATGCCCATGCAACTATAAATAGTTTATGCCTTCTCTACTTTTTCCACA
AGGCAAATACAAAACCCTAGTGTTCTACTCTAGTTAAAGTTCATCTCTCAAC
CTTATACTTCAATTCATTTCAATTTTCAACCCAATAATGGCTGCT

Glyma.09G122600

TAAAAACGTTTCAGGAAGTGTTTTGACCACTGGTAATCGATTACATCCTCTG
GTAATCAATTATCAGAGAGTAAATCTCTTGTAAAAACATTTTTGCTTAAATTT
ATTGGCCAAACCTCTTGCTGTTTCAACTTGGAATTACCTTCCTAAATCACTA
GAGATCTTCTTGATGATGTATCTTGAATTTCTTGGATTTTTGTCTTGAATTAA
ACTTGAGAAGTGCATGATCACATGACATCATCAAAACATCAAAGTCAAAGT
CTTTGCTTCTACAATACATTCATTTTTAAATAAACAAAAATTCTTAAATTATTT
ATTTATTGAATAAATTAATTCTTAAAAACCAACTTAAAAAATATAATTAATTA
AATAAAAAAACATTAGAAATATACAAAATGTTTATCTATAAATTAATAGCATA
AAAACAAAAATATTAATTTATTTTTAAATAAGAAAAATTTATTGTATTCTAAT
TTTATTAATTAATAAATTAAAAAAGATTATATGGATTGGAAATCCGTATAATTC
ATAGGGATTGAAAATCCATATAATTCGTATGGATTGACAATCCGTATGCATTA
TACGGATTTCGTATGCATTATACAGATTTCAAATCTGTATAAATGCCAATGCC
AAAGTAACAAGTATAAATGCCCGTGGAGAATGAAGAAGAGGGGCGCAGAT
GGAAGGAGTTACGACGAAGGCGACGTCGACAGTTGGGATAGTGACAATCA
CTGGTGACACGACGCAGAAGAAGTAGAGAATGAAGAAGAGGGAAGTAGA
ACAACATAGGAGACGAGAAGAGAGGGAGAACCCTTTTTAAAATTTTAATTG
AAGAGTAATTTAGCCACTTCACCTAAAATATTAGGTGCACTAACAATTATTC
TGGGTGCACAAAGAAAATCCCTTGAGGAAATTGGGAGCGGGTTCCTTCAC
AGCCACATAAAAGATTCCATTTCCTCTTTTCTACCAACACCCTCTAAACATG
AAATCATCTTACCTTAGCATGCTCTTTTTTTTTTTTTTTTTACGACGAATAGA
GATTGAGTTATAACATTCCTTAAAAATCAACAATTTATACGTAACAGTTTTTA
TAGTTTATTATATTAGCGATGCTTAAAAAAATTTATGAATAAAGAGATGCAGG
AAATGCATGAAATGCACGAACGGAAATATGGAAGGATAATATTATTTTTAAA
AATAAATAATAATTAAAAATTAAAAATATAAAGTGATTTGACAGAAATCACG
ATTCGTGCATTGTTTGCATTTTGCAAAACGTTATTCGTACCAATTACCATTTT
TTTATTATGTACGTACTTACGTTTTTTATAATAGTGGTGAGTTTAATTTTTCAA
TCAATAGTTATGACTTTGCACTTTATTTTTTAAAATATCACTATTGCTTAGAG
GAACCAACTAGTTTTATTTATCATAAAAATGTAAATAGATAATTTTATTTTAG
GAAATTAAAACAAAAAAAAACTGATCAATTTATGCACCAAATAAAAAACTT
GATTCCAATAGGTCCATTTCAATCTAATTAATTAGCTGAGTTTAGGATGCTTC
CTACTAACTAGGAGAAAAAACTAGTTTAGCTACAAACAAAGCACCGAATTG
ATAGTTTTTATTTCCTTTTAATTAAGTAAAAGTTGATTCTTCTTAACCACAAA
TAAAATATGGAAGCTTGAAATGTGAACATTGAACACTAAGCATATTCCCAA
GATGAATTCCGTTCATATTAGCAAATATTACATCATCTCATTAAGTTAGTTCTT
TATAATACATGACATAGTAGACGGACTAAGATATGGTTCATGCCAGAACAAT
AATCAAGCATTGTCCATGCTTTCTTAAGTTAATTAAATTAAATTATTCTTCCC
GCGGCCATCACCAAGTTTTAATATTCCATATTACCTACTATAGTAACCACTCC
ACTTGCTCTAGTTATTATATATAGCGCACACACACACAAATGCTTAGAATTCA

AGCATAGCAAAGATGGGAACT

Glyma.13G333100

ATTTGATATCAAGTAATTTTTAATATACTTAATAAATAATTTAATATTATTCTAA
GTAAATTATAATAATAATTTTTATTTAATATATAGTAATATCAACGAAAAAACC
TATTTATAATAATTATTAGTAAATTGTGTTTGATTAATTATATAGAAATAAAAC
ATTACATATTTTTCAGACCGTGCATAATACAAGCACGAAATCTTGTTTAATCC
TTAGAGAATAGAGAGAATAAAAAAAATATTTAGCTACACTACACGTTAAGG
ACAAATAACTCTCCTCACAACTTCATGGTCATCACTTCTGGCTGCCTCCTAG
TGGTTACTTAATTTTGAATTAGTTTTCTTTAGGTGGTATTCTCTGTATTGTAAT
TTTTTTTATAATAGTGTAACTCTCTTCCCATCAGATGAACCTATTTTTTTATAT
AAAATGATATCTCTAATTATCATTTCATTTTTAAATCGAAGATTAAACGTAGA
TTTTAGTTAAAAGACTCAAATGTTAAACTATTTGTGCCAACAATTTTTGTTT
CAGGCGATTGCAATATTTGAACTAAAGAAAATGGGACCTTGGCTTAAAATT
TTCGTCTTTTAAGCCGTCCATAATAAACAAAGAACGAAACAGATAAGCTTA
AAAATTATGATAATTTAAATACTGTTGGAGGTGGCAATGGGATTTGAAAATA
CTTGTGGCTCCTGATGCATCATACTGAAAGACAAATACAGACGAGTAGCAA
TTTGTAGATTATATTCGAGGCCCAGATTGAATTGATAGGCCTTGGCACTTTTA
TTATTATTTTAAGCAAATCACTTGAGCAGTTTGATACATGATTAGTTGATTTA
AGAATTAAAATCGAATCAACTGAAACCCTCATTTTCACAACACTACCTGCT
GAATTTTCAATTAGCTCTAACAAATCATTGAACATTGTCATAAAAAAAAAAA
AAAAAAAAACTTAGGCTGTGTTTGTCTGATCTGTAGTCGGATCCATGAAGA
AAACTAGTGAAACTTTTAGTAACAAATTTATTATCTTAATTGACGTCAAGTG
GCCACTATCTATGGACACAAAGTGATTAATTTATCCATTCGTAACAACTAAC
AAAATAAACATGACAATGTTATAGCGCAGGGACTCTTTGAATAAAGCATTA
ACCTTCGACCATCAAATGATCGTATTCACGTTGACACCACTCCAAACAACA
TTGTTATCCCACAGCTAGGAATGAAGCTGGCACACCTCCAATATCACAACC
AATAAGAGCCAATAATTTTGTGGGCCATCCTCACCTTTCAAATTCTGGAGCT
GACTTGAAAAAAAAATATCATTATTCATTAGACCTTTGCTCTTATCATCTCCA
CGAAACACATGGTATTCTTTACGATTGATTTTTGGTAACAAAAGTCGGAAA
AGATAAATTAATTAGTCTTTGAATAATTTGGTATTCTCTGAAGAGGAGTGCTA
TCAGCATTGATTGAAGGATCAATGCACCTAAATAAATTTATTAACTACATTTG
CAACACTAGTAAAAGACTGTAAAACCATATTTGATCTTATGAAAAGTAAAA
GAATAAACTAATCGTTACAGGAATTAATAATGAAACTTTGTTTGCAAGACAA
ATTAGTTGACTAGCTCCCTAGGGGGTGTGGTAATAACTAATAATAACTATGA
ATAATAATGAATCCAACATTATTGGGCCGGTCCACATCACCGCCCAGTAAAA
GAATTCAAAGGGTAGGTTTGGTCCAAAAGGTACACATTGATTGAAGGACCC
ACCAGCCCACAGGCAATTGGTCGGTGACCAGTGATTAGTCCACATCATGTT
GTACACGTGGCATCACAAGAAGGACCGGAAGGCCCGCCCCTCCGCCACCC
TAAGCAATAGACAGGTGGCAAAGAGTTTGAATTTTCTTCCTTATCTTTGTCC
CTCGTGTCTTAAACTCCGCTAGCTATAGTGTTGTGTAATACTATATAACACCC
GTAACAATTGCACAAAAGTTCCTAACAACGACTTAAGGCATTCTCTCTTCTA
TTCTATTCTAAACTCGAAACAATCTTAGAGAAAGAAGCAGAAGAAAATGCC
GATT

Glyma.14G089800

AAATCATTGTTAATTATCATTTTCATTATTATAATTATATAATTATAAAAAAAAT
CTAATTGCCATCAATTATTATAATTACATTACAATTATTATATAATTTAAAATAA
ATTGTCTTTAAAAAAATCAAATAAATCTATTACTTAGACCTCTTGAGAATGCT
ATGTGTAAACATTCCTATTGAAAAATATTTTTCAATATTTTTGTATGTATTATT
ATAGATTATATAACATGATAAATAATTAAATAATCAATAAAAATATGTGTAACT
CTTTAATTTCTAGAAAATTTATCTAAAGATTTTCAATGGTCAACCAAACAAA
TTTTATTCATCTCCAGGAAACATGATTCCCAGGCTTTGTGATTCCTAGGAAT
CATGATTCTCGGATATGAAAAAACTTATTTTCTATCAAACGTCTCGTAAAGG
AAATTTTTTTAAGTTAAAATTAGTTATCAATCATGTTGACAATTACAATGACA
GAAACAAGAATCTTCAAAAAATTCATTTATATTAAAAGGGTCTTGCTAACCA
ATGCCCTAAGGACATTGGTTAAGGAATTAAAAATAGAAAGTATTTATAATGG
AGATCATGGGCAAGCTAAAAAAAAAGTAATGGACAACACAATTTTGTATATT
CCAATATAATTTTCTTCCTTTTAATTCATTAACGAATGCCTTGAGGGCACTAG
TTAGCATTTGACATATTAAAATAGTTCAATAGAATTAAGATGTGCATATTTTTA
TAAAATAAAGCAAAAATAAAGTGTAGTTTAGACTTGACACTAGCTAAAAAG
TTAGCTACAAGTTGAGAAGTTAACGGGAAGTTGAAAAGTTAGTTAGTAATA
GAAAAGATTAAAAATAAAATCTAATAAATGTTCAAGGACAAAAAAAGAATA
AATATAAAAAGTTAAAACTGAGCGTTTTAATAAATACTACTTCAAGTAGTGT
CTAAAAAAAGTTAGAAGCTACCAAGAAATGAGCTGAAAACTATATAATAAC
AGAAAAATAATAAAATCATGATTTATTTAAAAATAGTAATTAGGAAAGATGG
TAATTATATTAAGAATAAAAAGAAAAGAAAATATAAAAAATTAAAAATTAGA
AACTAGTATTTTTAAAAATGATAGTTCAAGTAGTATGTCAAAAAATATTAAA
AATTATTAAAAAAAATTATTTATCAAATAATTAAATAAATTTTTCAACTAAAA
AAATAAAAATTAACTAAAATATCTGATGTAACATAATCTTAGACATTTCTTTT
ATGAGAAAATGAGGCATGTTGAAAAGGTAAAACATGACACACTTTCATTTG
TATGATATAATTCAAGGTCTTAAAAATAGTTACACTATCAACTAACTAAATGC
ATAAATTATTACTGTGATTCTCAGACAAATTCAAATGTATTTGCAAAGATAA
GCAGAAGCCTATATAATTTATACATTTCTAAATTTTAAAGGGTAGATAATAAA
ATCACATCACATTACGGGTAAGAAAGTGGGGATTTTTGCTGTGTACCAGCA
ATTTCAAACAATTACGTATGTTTATTTCTCTGTTCAGATTTGGTTAAAACATT
ATCTAACGTGTAACTTGCTTATTTATGGCCAAAACTTTACTCAATTCAGATCT
GCAAGAAACTTGAAAAGTAGTAGTATATGATATTAATATGCGAAGAAAAACT
AATAGGACACTCATGCTACAAAGTACCAAAAGAAGGCATAGCAACCACAC
ACACAATATATATATATATATATATATATATATATATTGCTGCTAGCTAGACACGAG
CACAACATGAGACAGAAATGCGACAAAAAGTTGTTTTGCTTTAATTTGCAT
GCATGAAAAAGTTTTCAGAAGCTGAGTTATGTAGGGTTTAAAGATGTGAAA
CGTGTACCGGATGGTTACTTGTTGGTCATCTAACTTTTGAATTTTATGTGATT
GGATTTGTCTGTTGAATTTCAAAGATGGTAATAATAGTAATATTTATAAGAGT
TTATTGGGGGTTGAAATTAAGTCTGGTTAGTTGACAATACAGAGAAAACTT
GCAAGGAACTATTTTCATTGGCAAACATCTCTAGTAATCCTTAGCTTTGTGG
TAGCTTTCTAAAAGGAAAACATTGACTTTCGTGGATAAAGGGAATTGTTTA
ACACTTCAAAGTTCAAATCAAAGGTGATTAAAATGTCTTCATTTGCTTCTAA
TGTAGATAGATAGTAGATTAGTTGACCAACAACGGTTCTGATTAAAGTTTGA
TATGAAGGATACTGTGTGGATAAGAAGTTGAGCTAACAATACTTCCATGCAC

CACCCCATTCCCACCAACTCACGCTGCATAGCCCATCATTGAGTTACACAGT
TTCGTCTATAAAAGTCCTAATATACAATACAACACCAGCAGTGTACGCTCAC
AAAACATCTCTAAACGCTTGTAAAGATCTCCATCTCCCATTGATCCTCCTGA
AGTTTTTGTATTCAACCATGGCTACC

Glyma.15G057500

ATATTACTTGAAGACGATTTTTTTACCCCCAAAAATAGGACGAATTTGATGT
CATTTATACTTTTAGAGGAATAATTTTGTGTATTATTAGTAAATAAAAGACTC
AGAGATGGTGTTACAAGTATCTTTAGAGGATCCAAAGAAGTCGAGGAGATC
TAGCTTAATTGATTGAACAAAGATATGTGAATTGTTGTAAATCTGTTGACAC
CATTTTCAATTCCTACAAATAAAATAAAAAAAAAAGGATCCAAAGTAACAGT
TACACTTCAAAGTTCAAATTAAAGAGCAGTTCCACATGTTGGATATGCGTTT
TAATCAAAACGTTCTGGCAATTTCAGCCTCCCCAGCATAGGTCGCTTCTCTG
AAAAGCAATATGAGGGATAATTCTAATCCCTGATTTAAGAATCCACCATACA
TAGGACAAGTTATTTCATGATTTCATTGTTTTTCCTAGAAAAAGCACAGTTT
TAACATATTAACATAACCTTTTTTTCTGGGCCCAACCAAAATTTGTTAAACC
TTTAAAAGGTTACATCTCAGGTCCAAATTGCATGACTTTTCAGGACAGATGT
TTATTTCTTCTAATGAATAATATTTTGAGAAGATTACATTCATCTTAAGATATT
TAAACCGTTGGTAATGGAATTTAAAACAATAATGTTTATCTCAAATAAGAAG
ATTATATAGGTGCTGATAAACTGCTACAGTATAATTCATAGGAATGTTATTTGT
ATAAAGATTTAAATTTCAATTCTTATTAGTTTTATTATTAGTCATTTTATATATA
TATTTTATTTTTTAAAAGTACTTTTAATCTATTGACTATCCTTCAAAGAGGAA
TTAAAAACACATGATTTTTAATCCATCACAAAACATGCTAAATCATCCATTAT
ATATATATATATATATATATCACTGAAAACACGAAATTTCATAGAATTTAACAG
ATCTTTCTTTGTCGCATACTTGAATAAGTTCTGGTTAACGAATGTCGCTCTCT
GTGTTGCAGAGCAGAACATGTTACACATGTGGCTCCATTTGAGGTTAAAAT
CATGATAACTTTAGTTACAGCAACATTCATACATAAGTGGTCCACTGAGAAG
CCGTTCTTGTTGTCCACAAAGGGAAAACATTACCTTACTCTATTCTTGTGAT
TCATAATTCATTACTCACTGGACTCGCCGTGAGGGTTTGAACCTCCAATACA
ACAAATGTTTCATCCTCTCTCAACTAGTAAAACAATAAACAGAGTTGCGTTT
TTTTTACACCCTAAAATTGTAAATAATTTTATATGTTATTTTTTTTAAATAGTT
TCTAACTAGTAATTAATTTCTCGTTAAAAATTTATTCAAAATAGGACTTTTAA
AAACACACACAAAAAGAGAAACAATTAAGAGATTATTTCTCGTTCTCTTCT
TCATGACGCTAGATCAAGAGTGTTGGTCGTCCAGTAGTTGTTTTTTTTTTCC
TTTATAAAAGTCGAGTTCATTGTCACGACTTATTTAAGCGAAGTGGTCAAAT
GAAATACAACTATAGTTTATTTTCTTAGAAAAAATATTAAGTGAAGTGGCGA
ACTTAAATAATTGGAGACTCAGAAATTAATTAATATAATATAATAAAATTATG
AAAGTTTTAAGAACAAAAGAGTATTTAGACATTAATAATATCAACTCATAAC
TCAAAAAACAAATTCCAATATTCCTCTTTTTTTTCTTCTTTCTATTCTCAAAG
TTGGAATACCAAATACATTGTAAACCCACAAGTAAAAATCTACTTTCCCCCC
TTTTATTATAAATTATTCTAAAGAAAAAAAAAAGTTGGTTGTCCTACCTTTGA
ATCCCCAAGATTAAAATTAAACTATCCAACCACTACCAACAAGGTAGTCCA
ACAACGTATTGAGCAGTCTAAGGAAGGTTAGCAACCATTAAGAAAACTCA
AAGGGACTTAGCCACAAAGGGTTGCAGTAATTTTGTATAAAAGGCCTGCCA
CTAGCATCTGGAATATGCACACATTAATTAAGCTCAAAGCAAAAGTCCCTTT

GGCCTTTGCAAACTATCTATCATTTATCAGTTATCACAAATTAAGCAAATTAA
GTCTTTTGACATGGCCTCC

Glyma.15G251300

AAAATTCTTATTTGATAAAGATTTTTTACTATACTTATGAATTCCATTAGATCC
AAAAAGTATACATTGGAAGAATTATCTAAGTTTTCCAATATCAATAGGTTGA
TTGTTTGGCTTCTGTATTTTCTAAAAGGAAAAAAATCTGAGATTCTTATTTTA
TAGTTTTTAATAACTTTAAACCACTTAAAATTAAAGCAATATATTTAAATGAG
TGTTTAAATAGATAACTTGAGTGTTGATATTTGAAGTTTGAACACATACCCA
CAAATTATAAAAATTTTATTAATACCTTCTATTTTTTTATGTTTATCTTCCTATC
ACAGTATATTACTAATTATATCAATTTTTTTTCTTCTCATCTCTCTCTCAAGAT
ATAGATACTCAAAAGTCAAAGACTGTCCAATAATCATTTCAAAAAAAACTT
TAAAAGACACATGTTGAATATTTTTTGGGACTCTGTTTAAGTTCATCTATAGA
TGAATTTAGTTTCAGTATACCTACACGTGGTGATACAGACACCCCTAAACTT
ATTGATGAATAGAATAGCATGTGATGAAAAAAAAAATCATTATACTCTTGTC
TGATTATTTTATGTTACTCTCCTATGTTTTTCTATTGAAATGTTTTTTCCAAAG
ATCTTCAACTGTTGACTTGCATGCTCCATGCCGCAGCAGGACTACAAGCCT
AATTAACATTCACCTTCTCGTATTAGTGAGTTAGTGTGAACACAGATATGCT
TCCACCTTCTCATTCTACAGAAGGTGTGAAAAGAACCAAATAAACTTAAGA
TACCATCACTGTTCATTCATTTCCGTGAATTTAATTAATAGTGAGAGACTTGC
AACATCCACACGCTTGAAGTTCCAGTACTACCCCAAATAGACAATTTTTTAA
TCAATGCTTTATCACCATTAGGATTGGAAATATAGACTGAATTAAACTAAAG
ACACGCAATTGTGTTAAATTAAATATTCGAAAAAATAACTGTCCATGTTGAT
CACTAAACTTATTACTTAAACTTTATAAGATTTGAACCAAATCTATTACTTAA
ACATAAGGTACGAGTCTAATCTATTTATTTGATATGGTAAAAATTAAAATAAA
AAAATGTAAAACCTTTTAAATAGAACAAAAAAATATTTTAGTAAAAATCAAT
AATAATAATAATAATAATAATAATAATAATAATAATAATAATAATAAAAAATGT
TTAATGAGCATGTAGTATAAAAAATTTATATACAATCACGTAGTAAAAATTAT
TCTTAGTATAACTTTTAAAATAATTATTATAAAAATTAATAAATTTATCATATAT
AATAATTTATAAGTGGATACCAACGTAATTATACCATTGGTGTGGATTGTATC
TTTTATTTATTCAAATAAGGCAAATGATAATTAGCATCACACCATTAAAGAAT
AAAAAATGAAACTTTTTATTAAAATATATTATTCCTACCATAATTTCCAATGTA
TGGATAGCTAGCATAATTTCTAATAAAATCTCTAAATATATTAATTTCTTAATA
CGTACCTTTAGGATACTAACTATAAAACCATCATCATCATCATAGTTTTAAAA
TAAACGGACTTATTATTTTTTTTTTAAAAAAAAGACCAAGTTAACCTGATAT
TTCAAATTAAATAGACTTTTAAAAAAGAATTAACCTGAACTAATTAGTAAAT
AGGCCTAAGTTGCTAGCTTGGGCCTGAAAGCAAACTTAGACTTATTTTCTAT
CTTCCACCGTTATTTGTGATGTATATATTTTGTACAATTCCAATATTCAGATGA
TGATTAGATAGATAAAACCTATCTATCACCATCATGTATCCAGGTGATTACAA
TCTTGAAGGCATGAAACACCCCTTTGTAGTTGACCAGTACATGGATTTCAG
AATTCCAACAACATTAATTAAATAATGTCCAACAATAACAATAATGACCTTT
CATTCCATGCCCATGCAACTATAAATAGTTGACGCCTTCACTACCTTTTCCCC
AAGGCAAATAACTAGAAAACCCTAGTGTTCTAGTCTAGTTAAGTTCATCTCT
CAACCTTATACTTCAAATCATTTTTCAACCCAATAATGGCTTCT

Glyma.19G173800

CTTTTGTGTTGGTCTGTTATAATGCTTTTGTTATGCATTGCTGCCAAAAATCA
TCATTGGCAATAATGCATGACAGGACATTTTCATTTTCAATTATTTAGATAAT
TAACCATCATTAGAAAAAATTGGTGGTTTTACATTGTCAATTTTGATTTTGTT
GTAGTATTAAAGAACCTAGAGCTAATAGAGTTACACAACACACACACCCAA
GCATTTGATGTAGTGAGATTAAAATGATGCATTGTATATCTCTAAGCATTTAA
TCCTTCAACATGCATATGGTATGTCTCCAAGCATTTTATGCATCAAGATTAAA
ACAATGCAACTAGTATCTACCATTATAAGGAGTGTCAACACAAATTGTGTTA
CTGGTAAGCTACCCCGTCTTGTCTAAGAAGTTGTCTGGACTTTGTAATAACC
TCAAGACGTTCTGATTTATGACCAATGAAAATTAGTTGAGGTAAGAGGTTTT
CAAAAAGAATACAAAACTCACAAAATAAGAGGGGGTGTTGATGAGGGGAA
AAAAGAAAAGAAGGAAACAAAGGGGACAAGAGAAGAAGGAGGAGAAAG
AAGAAAGGGACATATTTTTTATAGGGACTAAAACATAAAAAAACTCATATAA
GGACTGAAACATAAACATTGTACACTTATACGGATCAAAACATAAAAAGTA
CAAAAGTATAAGGACTATAAATGTAAAATGCAACACATGTCATATTAGTGTC
ACATGATCCCCGATTAATGGTGTTAAGGTTTTTTTTTTTCACTGCATACTAAA
ATAAAACTAAAAGTGTTTGTATAAGGCTAAAACAAAATGATTTTTAAGACC
AAAAGCAAAAGTTCATGTGATGGTAGGAAACAAATGAGTAATTAGACAAAT
ATATATTTTCTGATATGTTTGTATATCTTCTGTATTAATAAGATCATGTGCATAC
ACACATCTACAATCAAACCATTCTAATGTTCCATTTTAAGTCTCTTTAATAAG
CAAAGACAAACAGTAATTTTTTACAAAATGAGTAGGGGCACAGACCCACTT
GTGTATATATAAAATAAACTATGAGTGGGTACTGAGAGCAGAAAGGAGGTG
GAGATCCAAATAGTCATGCTAGTGGCTGCACATATGAAAAGCACAAAGATA
CAACCGCCTTAAAAAGGCTTAATTAAAGCCACCAGCTACAAAGTGGACCT
GAGTGCTTACCATACTTTGAAGTCTTGACCTAAGACCAAAGTCACCAAACC
CTTAATGCATTACTGTGGAAAGCAAAACTAATAGCCGAGGATATGGCTATTA
TGCTTGTGCTAATCATCCTTCATACGTCGCTCTCGACTATATTGACTCATTGG
ACTATATTCAATAATGCACTTGAATAATTCACGACCCTTTTTATCCAGAACTT
TAGATTATTTTATCCTTCCATTAACTCCTAAGAGGCTAAGAGGAACGAAAGT
ACATCTTCTGCAATTTGCAGAAAATCAGAAATATACAAATAAAAAGGATATT
TGTCATGCCAATATCAGAAATTTACGTCCAATTGGTGCTGTACGTCACATAA
TCAAGAAGATCAGGTCCATCATGGATTCTAACTCTTTCTTTAAAAGTAAACA
AGCACTTCATCTGAAATCATCTATTGGGGCATTAATTACTTTTCCTCATCGTA
TGACATCACAGAGAAAATTTGTCTGGAGAAAAATCTTCATAGATATATAGTA
TTAAGTAAAAATCAAGTAAAATAAACGAATCATGGAAAAAACAAATCAATA
AAGAAGAAACAAGTGAACGATATTATAAAAGGTACTCCATGATGAGTAACA
TTCCAATTATGCAAAAGATAGTAATAATTGAAAGTAGACACCCACATTTTAA
AGAATTTGAGGATGAAGTACATTGATTACTGTGACTCTCCCCCACTTTGGTT
ACGTGAAATAGAATTGCTCTTATTTGTGGGTATAAATATAATGTATAATATACT
ACTAACTTTCTAAGCACTAATTCATTATACACCACAAACCCCTATATAAACA
CGAGCCTTCTGTGGTGCAGCGGTGTGCTACTTATTCTTGTGGCTCAAGAAAT
AGGTTAGTTCCGAGCTGAAACAAGATGGCTGGT

Glyma.18G211000

AACTAAATTCATATAAAAGACACTTTTCATTAAAAAAAATTCTTAATAAGAG
AAAAAAAGTTGAATTGAAAGTAAATAAAAGAAAAAGTTTTTCAATTTTTAT

TTGAGTTTTAATTTCTTTTTTATATCTATTTCTCTTCATGTTCTCTTTAGTCACA
TAAAAAAATTCAAAAAAAAAAAAATTAGTTTTACATTTTTTTGTCAGGTTTA
CATGTTTTTAAGTTTGTGGACTAAATTTGTCGATGTAAAAACTTTGATAATG
TTTGACCATAAGTATGTGCACTAAAAATACATTTTTTTCTAATTTTTTTAATA
AAAAATTGTCATTTTCTTTCTTGAGATAGAGAATATGTAATTGTGGGAAAGA
GCTGGCTGCAACTGCACTAGTATGCATATATACGTTACTTTATTGACTTAGCT
TCCAAGTAAAGGATTACCTGTGTCATAATTCAAAAGTAAAAAGATGCGTAC
AAATTAAAAAAATGGTAAAGACATGCTTTTTATTTTTAATTTAAAATAAAGTA
TTTCATAAACTCAACTCGTTTTAAATCAAGTGTTTGCTAATACATATAATTTC
ATTTTTTCTAACCTTAAATCATTTCTCATTTAGAGGACTTATTCGTATGCATG
GTTATGAAAAGTACTATTTGCACGCTCATGGTGCGTACGCGTTATTCGTCAA
AAAAGAAAGGCAAGTAGGTAACACGTTTGATTAACGCAGAAAAGCCAGAG
AACATGTTGGCTTGATCATTGACCTGCCTTACTATCTTCTAACAAAGCATAA
ACACTTTCAGACGTTTGTTAATTATCTGTCACACGAATCTAGCTAGCTTAATT
TCATCACTTTGGAATGTTTTTAGTTGACAATTTTTTTACTCAGAGAGAGATA
ATTAATTAATGATTTTCTAAAGAACGGCATAGGGAAAATTTTCTCCTACAAT
AATACCATAAATTACGTTCACTCGCTGTCTTCAAACGGTTTCGTCAATGGAT
AAATTATTGGTTCATCATCTTCATTTTTATTATATATGTATTCAGAGAAAAATG
CTTATCGATGTATTAATGAAACACTTAAATGTGGACTCCACTTGACTACTTC
AACCCACTTGTGAGATTAAAACCGGCACCACATAAACGAAACTGCAGAGG
GGCGAAATGAGTTTGGGTCAATACGTTCCACTTTATAACATTTCACACTAAA
AAATAATATATGTGATTAAGTTGATTGTAATTCACTACACAAGAAGAATGAC
TTCACGAACAAAATTTGTCCCCTCAGAAGTCAATAATCCGTATATAAAAGCA
TAATTGATATTAACCTTAGTCTATTCGTATAAATTTTACCTACATATATACTTTT
ATCTACAGTTGTATTTATTTATCTCTCAGTACATACAGTTTATTTTGGATGTAG
CTAAATATCATTAGCTTTTACTTACAATTTCCCACTCTAGTTAAAGGTCGCAT
TAATAAAAAAAAATGCAGCTAAATTCAATATATAGAAACACTAAGATATTATAT
TGAAATTTTGTATAAACTAGTAAATTTTTTATAACAAGATTAACTCGATTTGG
ATATTGAAACGAAAGAAAACGAATTCATTATAAGGATGCATTACTTCTAACA
TATATATGTCTTGGAGAAAACATGCTCCTCATGTGTTTTTCTTAATAAATTAA
GAATTGTTAAATTTTTCATGTAAAGTGGGTCGGTCTCAAAAGCCAAGAAAG
AAAAAAAATACACAATTTGCAAATTTTTCGCTCAAATTAAAGGGACATTCT
CTGTAGGCTTTAGTAGAACAGTCTCCAAAGACTTTTAACAAGTCAAAATAT
TTTAGAAAATACAGCTAGCTTCAGCCAAAAGTTAATATTCAATGAAAATCAA
CGATTAACGCAGATAATACCTACCATTTAATCCATGATCATAGCTGCATATATT
GACAAAGAGAAAGTAGACGGACAAATCTCATATAAATAGGTGACAATTTCT
TGGTATTCACCACATTTAAACACTAATCATAAGCCTCTCAACCCTATCCTTCT
TAGTTACATATTTCAATTTTAAAATTCCAATTACAATGATTCTT

Glyma.20G022500

TTGACTATATATGTTTGATAAGACATTTAAACTAGTTTATAAATTTTTTAAGTA
GTTAAAAAACTCATTTGATTATTCGGAAAACAAGTTTTTTTTTTTATGGAGT
CTAGCGTTTGTTGAAATGTTATTCGAAATAACATTTTTTTAAGTTTTTTATATT
TTTCCTTTTTATCCTTAATATATTTATTAATTTTTTTGTTTATCCTTTTTAAATAA
ATCATAATTTTATTATTTTTCAGTTACTTTACATTTTTTAATTACTCTAACAATT

AATTTTTTCACTTATACTTTAAGTAGTTAGTTTTCAGCTTTTTGCCTACAGCT
AACTTTTTAGTTAATTTTGTTGAACAATCTTTACATAATTATCATTCTAGTTAG
TTTTGAATTCTTATAATTTTGTTCTTAAAATCAAATGTTCTTTTGAAAAAAAC
AACTAATTATTATAATTAACAGGGTAATATTATTTTTATAAAAAAATATGCAAT
ATTAAAAGAGGAAGGAAATTAGAAAATTAATTAAAATATTAAGAATAACAAT
AAAATCTAGCAGTAAATTATAGAATCAAAATACTATTTTAAGAATTATTATAA
ACTAATGAGATAAATTTATTTATTAAACACTTTATTTACATAGAATAAAGTTCT
ATAACAATACAAATATTGCAAGAAATTCCAAGTTGAAACACTATTAAGTATT
GTAAGCTAATAAGACAAATTCATTGAAAGTTATGTAATAATAATATAAAATAA
TTTTATATACTCATTAAAATCACACCTCAACATGATAGCAAGTTCGTTGATCT
TTAACAAAAAAATATTTTAAAAATCATACTATATATAACCATTAATCTCAAAT
TCGTTTGTTAAATATTTTATTTCCATTGAATCAACTCATCTTGTTACACACAC
GGCCACAAGGACCTCTTGGCAATCGACAAGGCAGAGATACCTGTGCCGTAT
CACCTCCATGATGATCATCACAAACAGCATGAAGCCATCATCAATTCAGATA
TTTTATGCTTGTTGATAAGGGGCGCACGTACGTCACCAAGTTTACTTCTTAA
TGACATAGATTATAAAAAACCATAAACAGCATGATCCATGAACACTTCAATT
CTTTGTTCATTTTATGCAAACGTAAATTATTTCTACTTGGCTATGTGTATGTGA
TATGCATTGACTTCCCTACACATTTGTATATAAAATTATTTTCATCATCCTATT
AGAAATAATTGGTGATCCTTGCCGTAGTACCCATTTTCGTACGGTTAATTTTT
TACTATTAATATTAGTCCTTTTTATAATTAAAACTGTTGTGCAGTCACATTAGT
TGGCGTTATTCATCCATCGTGGAATAATTTATCATGTGTGCACTTGTTTACTG
CTAGTAATTGGGTGAATACCCAAATTTGTCCTCAACAATGTGAGGCATCGTC
ACTTTAATCCTTAAAGTATAAATTTTTTTAAAAAATCTTGAAAATGTAATTTA
TTATTCACTTTAGTTCAAAATTTAAAATATTAATTGAGTGTGTATTACTTAAAT
AAGAAGGATTTAATTATTATAGATTACCAGTGTAATTTTTTATACTGTCAGCC
AATAATATGTGTAACTTTTAAAATAATTAGGATAAAATTCAAACCTTTAACAT
GTAAGCTTTTATTACTTTATTAATGCATACCAATTAAATCCAAATTAATAATAA
TATCAGCATACTTTCATTGATTCAAGCAATAACAATTAATTAAGGAATTATAA
AATGATAGCTTATGGAGTTGTTTTGAGTTTCTTACTTTTAGAGACTAACAAT
GCTTTATAAATTCAGGAGTAAATTAATAGTTTGCCGGCCACTATTAATTCTTG
AATGAAAACAAACAAGGCAATTGTAAAGTCTATATAAAGAAGCTCTATCAA
AAGCAAAATATACAAGTACAAGTGAGAAGTGTCCTTTAGTTTGTGAAAGAA
ACAACTACAAACAATGGCCACT

Glyma.20G007900

AAGAAATTAAAGTTTGACATAGTCATTTTGACAAATGGTATATATATCTAGAC
AATGTTTCTATTAGCACCCTATAAAGTTTATTATCATGGTCGTTTCAACAAAT
TTCCTCTTGTCGGGTCTGTTTAATTAAGATCACTTCAACCATCTGTTGAATTA
AGATGTTTCTACTTTGTTTTTGTCCTTTTGGTGGATAAGCTATGTACTATATT
GTTGTTAACATAGTCACGGCACCTAATAAGTTTTGTTACACCTATTTCCAAC
TGCATGCATCAATTATTCCATTAATTAAACCTTGCCCAACTAACTTGCAAATC
TTTTTTTTTTTTTTTTCCTTCTGTGGTTTACTGCCCCCATTGATTATTTCTGTT
CTAGATTCATTTTTCAATCCAAATTTACCAACTCATTAAAAAGTTACAAATT
GGCAAATGGGAAATCACGGGGAATTAAGAATGCTGGAGACATAAAGTACAT
TTCACACAGCTAGCAAATATTAGGATGATGCAAATATAGAAGAAAAAGAAA

ATCAATACATGTATTTTATGCTGAAATTCTTAAAACTTGATTTCTGGAGTATA
GAATGAAAAGCCGGCATTGGGTGTATGACTTTCATCGAGAAAATACAGAAC
CAACTAAGTATTCATTATTCAATTAATGCGCGTCTAAGACACCTTCGTTATCA
CTGTTCAGTGTTGACTAAAATTAGCAAGAGCAAGATCATGCATGATTTCAG
GGTGCATTATTGGCCAGACAAATGGCTTCCCCATCAGTAATGGTGGTCCTTA
ATAGGTATGCATTAAACGTAGATAGAGATATTATATGTAATTTTAATCAAAAC
TCTCCACTGTCATCACATTCACATATACTAAAATATATTTTTATGAAAAAATAT
ATTTTGTACTAAGATTTCTATACATGGGACGAAGTCTATAGCTTTGGTTTCTT
TCCAAACAATAATAACAATAATAATAGAACAATACCATTTTAAATTAAGAATT
TAATTTTAATGTATGCAAATTTTTTTACACTATCAATCTATTAAATATGAATTT
TACATTAATTATTATAATAGTCATCGATGCTTTCTAGTTAAATTTTGAAAATAA
TAATTATGTGATATAACTTCAAATACTCCTACCGGTATTTTGTATTTTTAAACT
TTTATACTAATACAAATAAAATTTCATAATTATCAGATAAAATAATAATATATTT
ATGTATTTTTAATACTCATTTCCATTTTTAAACAATCAATACTTCCTTTAGTTA
AAATTTATAGTTATTGTTTTTTTACACAAGTATTATGGTTGTGTTGTATATTATT
AATATAATGCTTCTTAAAATATAATTGGTAGATAATTGAGTTTATATATTAATAT
GCTGACATGGTTTGGTTACCTGATCAGGTGTATAAAAAAGATTTATTTGGAT
AGATAAAATTTGCTTTAATAATCTTTATGTTTAGAGAAATTAATCTTATGCCTT
TTAGTAGGAGAGTAGCTTGCTTTTAGAAAGAAAAAAAAATTCTCTAATTAG
AAAGTAAGAGAATAAAGAAGTTAATAAAGTTTGATTTAATTAGTAATAGTTG
AGTCCATATCTCATAAGGATGTGAGTTCATGATATCCCATTATCAAGATGATG
AGTGATAACATGTTGATAGATAATCTATTATCTATAAATATCTTTGCGAATGAT
ATGTTCCTTAAGACGTGTCTCAACTTTAATGAATTTTTGAGATCCAATTTATT
CATATTTTTGAAGGGCGGGAAAAAAATAAATAAAAAGATAAGAGAATAAGT
GATGAATAAATACCCAAAGTGGTGCAATACAAAAAGAATAGAAGAATAAAA
AAAATGAGAAGAAAAACCAGAAAGAATAAAAAGTAGGTATATGTTAAACG
ACATTTTCCTTTTCAAATTAGAACCAAAGTTTATAGGCCCTTTATTTTATTTT
CTGTTAACCATTTTGTCTATTAGGAAAATGGCAAGGGTAGCTTCACCAAACT
TTTGCAGCCAATATAAATGCTCATGGAGGGTGTGTTGCTGAAAATATGCAAA
CACAGAAATCACAGCTGAAAGAGGGTACTATCAATAAAACAGCGCTACAG
ATATTGTTCAGAAATTTCTGTACCAAAAAAGTAAACATTGAATTTATTGCAC
TGGCTCAACCCCCCTCTTCCTTTCTGGCTCACATGCACTTCTTTTTCTTTCTT
TGAAGTTCATCTATCCACACATACATATCCCTTTAGCCTTTATTACAAGCCTC
AGATATAACTACTTCACTTGTTGTGACTAAGCTCACTAATCATTATCATCATT
CATCATCCCCTAGACACAGTTCTGAAACATTCCATTCCATCTTACAATGCCA
GAG

2. Supplementary Figures

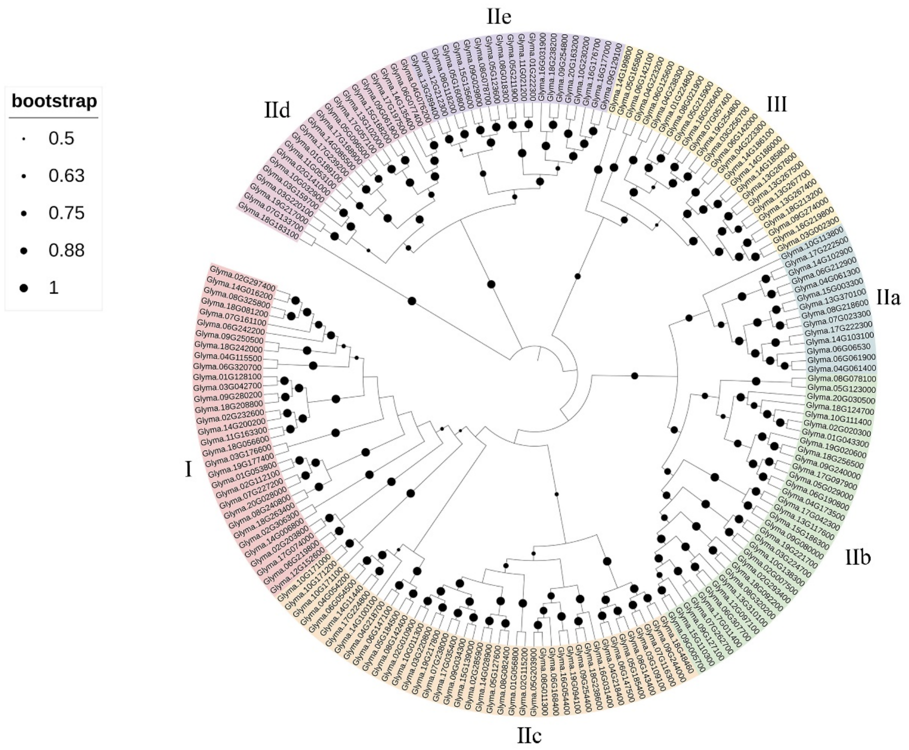

Figure S1. Phylogenetic tree analysis of 188 WRKY transcription factors members in soybean. The size of the black dot reflects the reliability of branches, and different groups of the WRKY family are shown in diverse colors.

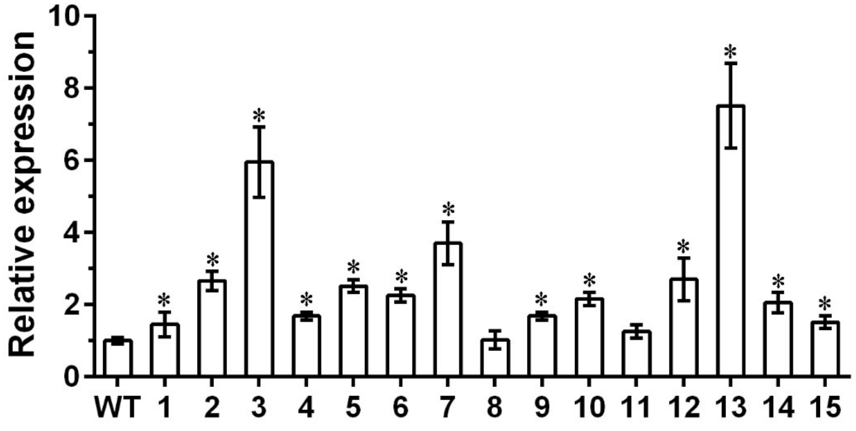

Figure S2. Quantitative real-time PCR analysis of overexpressing GmWRKY81 soybean lines. Data are means ± SD of three biological replicates. The * indicate statistically significant difference, using one-way ANOVA and Duncan’s test ($P \leq 0.05$).
